# Notes and News

**Published:** 1896-10-17

**Authors:** 


### NOTES AND NEWS.

(Collected and Communicated.)

Mr. John P. Bryant has been elected secretary of the Central London Ophthalmic Hospital. Mr. Bryant previously held the post of collector at Charing Cross Hospital.

The Home Secretary has appointed Mr. Thomas Pickering Pick, F.R.C.S., to be the Inspector of Anatomy for the Provinces, in the room of Mr. John Birkett, F.R.C.S., resigned.

The latest news from India record the outbreak of bubonic plague as of a mild character. At the beginning of the present month about eleven deaths occurred daily in Bombay.

Referring to an article which appeared in The Hospital on October 3rd, urging instruction of the people in regard to vaccination and its power of controlling small-pox, the secretary of the Jenner Society writes to us saying that it is the diffusion of useful knowledge in print rather than in speech that is most likely to be useful. This, he says, is the object of the Jenner Society. But this, we may add, is just what we do and have done, in number after number, for a long time back.

Although the programme of the Hospital Reform Association has not yet been issued, we may state that it will probably aim at promoting reform by making representations to those hospitals where abuse is known to exist; by arranging for interviews with the managers and the medical staffs of such hospitals; by drawing public attention to the enormous growth of the out-patient departments, and suggesting remedies, such as a wage limit, the appointment of an almoner, and the limitation of the number of new cases; and by other measures. No one can reasonably object to such a programme; much, however, will depend upon how it is carried out.

The Croonian Lectures will be delivered next year by Dr. Hale White, and not by Dr. Sydney Martin, as announced. Dr. Martin will lecture in 1898.

A bazaar will be held at Cardiff during the first week in November in aid of the Cardiff Infirmary, which is burdened with a debt of £7,000.

The Medical Service in State on St. Luke's Day, announced to be held at St. Paul's Cathedral for the first time, will take place in spite of the death of the Primate, who was to preach. It is now hoped that the Bishop of Winchester will be able to fill the pulpit on the occasion.

The late Archbishop of Canterbury took the keenest interest in hospitals and charities. He was connected with upwards of twenty-five institutions in an official capacity as president, vice-president, or patron, and exhibited great discrimination in centring his interests on those most worthy of his patronage.

Mr. Thomas Bryant, F.R.C.S., has been appointed Surgeon-Extraordinary to Her Majesty the Queen, in the place of the late Sir John Erichsen, Bart., F.R.C.S., an announcement which will give universal satisfaction. We hope this recognition of Mr. Bryant may be followed by yet greater honours in the near future.

A correspondent of a certain newspaper, possibly to make copy, reported the death of a promising student from diphtheria, an immediate result of overcramming. The connection between education and diphtheria is novel, and we trust it may not form a screen behind which the managers of insanitary schools and colleges will protect themselves!

Our attention has been called to an appeal issued by the notorious Queen's Jubilee Hospital, Earl's Court, S.W., in the course of which the committee have the effrontery to remark that this institution is "the only free general hospital which is situated in the midst of a large and indigent population." We have pointed out on more than one occasion that this institution is not a general hospital at all in any proper meaning of those words, and that those who have money to distribute for hospital purposes would be wise to devote the whole of the money to real hospitals of approved public utility.

In the October number of *Science Progress*, which is now issued in a somewhat enlarged form as a quarterly, there is an interesting article on "Scientific Weather Forecasting," giving a description of the method by which the meteorological forecasts, to which we are now all accustomed, are drawn up. Long ago, before the days of telegraphs, the progress of storms had been observed, and comparison of observations had showed that they obeyed certain laws. This, however, was of but small practical use until the introduction of the telegraph made it possible to construct storm maps, if one may so call them, while the storm was actually going on, and thus to give warning in advance along the track of its progress. But the facts in regard to storms were known long before any daily weather chart was issued; the power of forecasting the weather being dependent not so much on increased knowledge as upon the rapidity with which the facts can now be accumulated by aid of the telegraph.

